# A recessive lethal chondrodysplasia in a miniature zebu family results from an insertion affecting the chondroitin sulfat domain of aggrecan

**DOI:** 10.1186/s12863-018-0678-8

**Published:** 2018-10-11

**Authors:** Ann-Kathrin Struck, Claudia Dierks, Marina Braun, Maren Hellige, Anna Wagner, Bernd Oelmaier, Andreas Beineke, Julia Metzger, Ottmar Distl

**Affiliations:** 1 Institute for Animal Breeding and Genetics, University of Veterinary Medicine Hannover, 30559 Hannover, Germany; 2 Clinic for Horses, University of Veterinary Medicine Hannover, 30559 Hannover, Germany; 3 Department of Pathology, University of Veterinary Medicine Hannover, 30559 Hannover, Germany; 4 Veterinary practice Oelmaier, Rot an der Rot, Germany

**Keywords:** Cattle, Chondrodysplasia, Miniature zebu, *ACAN*

## Abstract

**Background:**

Congenital skeletal malformations represent a heterogeneous group of disorders affecting bone and cartilage development. In cattle, particular chondrodysplastic forms have been identified in several miniature breeds. In this study, a phenotypic characterization was performed of an affected Miniature Zebu calf using computed tomography, necropsy and histopathological examinations, whole genome sequencing of the case and its parents on an Illumina NextSeq 500 in 2 × 150 bp paired-end mode and validation using Sanger sequencing and a Kompetitive Allele Specific PCR assay. Samples from the family of an affected Miniature Zebu with bulldog syndrome including parents and siblings, 42 healthy Miniature Zebu not related with members of the herd and 88 individuals from eight different taurine cattle breeds were available for validation.

**Results:**

A bulldog-like Miniature Zebu calf showing a large bulging head, a short and compressed body and extremely short and stocky limbs was delivered after a fetotomy. Computed tomography and necropsy revealed severe craniofacial abnormalities including a shortening of the ventral nasal conchae, a cleft hard palate, rotated limbs as well as malformed and fused vertebrae and ribs. Histopathologic examination showed a disorganization of the physeal cartilage with disorderly arranged chondrocytes in columns and a multifocal closed epiphyseal plate. Whole-genome sequencing of this malformed Miniature Zebu calf, its dam and sire and subsequent comparative sequence analysis revealed a one base pair insertion (*ACAN*:c.5686insC) located within the cartilage development gene *aggrecan* (*ACAN*) exclusively homozygous in the affected calf and heterozygous in its parents. This variant was predicted to cause a frameshift (p.Val1898fsTer9) and thus a truncation of the chondroitin sulfate domain as well as a loss of the C-terminal globular domain of ACAN. It perfectly co-segregated with the lethal bulldog syndrome in Miniature Zebus.

**Conclusions:**

We found a novel mutation in *ACAN* causing a recessive lethal chondrodysplasia in Miniature Zebu cattle. A diagnostic test for this mutation is now available for Miniature Zebu breeders preventing further cases of bulldog syndrome by targeted matings. To the authors’ best knowledge, this is the first case of a Miniature Zebu associated with an *ACAN* mutation.

**Electronic supplementary material:**

The online version of this article (10.1186/s12863-018-0678-8) contains supplementary material, which is available to authorized users.

## Background

Chondrodysplasias are congenital malformations first described in cattle in the early twentieth century [1]. Generalized chondrodysplasias in domestic animals were designated as bulldog syndrome [2].

Characteristics of the bulldog syndrome are a premature termination of metaphyseal bone growth due to a disturbed endochondral ossification leading to a shortening of cranial base of the head and spine, a premature fusion of the spheno-occipital joint, a limited length growth of the tubular bones and consequent constriction and contraction of the limbs [3–5]. A disturbed endochondral ossification in bulldog calves primarily affects the growth of the long bones [6]. Bulldog calves exhibit micromelia, short spine, large abdomen, and a relatively large head. The head shows a shortened nose, cleft palate and a protruding tongue [7, 8]. This anomaly has been observed in several cattle breeds including Dexter [1, 3], Holstein Friesian [9–12], Miniature Scottish Highland [8, 13, 14], Miniature Belted Galloway [8], Nellore [15], Charolais x Salers crosses [10] and in Nganda cattle, a small Zebu crossbred [16].

The mode of inheritance for the bulldog syndrome in Dexter cattle was proposed to be incomplete dominant with a lethal bulldog phenotype in a homozygous state and without abnormalities but shorter legs in a heterozygous state [7, 17]. Breeders of Dexter cattle observed more often calves, which were aborted before the seventh month of pregnancy with an extremely marked and constant type of deformity of the body [3].

Two mutations were identified in the *aggrecan* (*ACAN*) gene on bovine chromosome (BTA) 21 for the Dexter bulldog phenotype. An insertion in exon 11 (BD1) and a second variant in exon 1 (BD2) were identified in bulldog affected Dexter calves [17]. The BD1 mutation was also found in bulldog phenotypes of Miniature Scottish Highland and Miniature Belted Galloway [8, 13]. In these breeds potentially derived from intermixes with Dexter cattle, the mode of inheritance was also suggested to be incomplete dominant [7].

Very similar bulldog phenotypes caused by *COL2A1* mutations were found in Holstein Friesian cattle. Four different missense mutations in the *COL2A1* gene on BTA 5 were reported where each one is responsible for bulldog syndrome in Holstein calves [9–12]. Affected Holstein Friesian calves showed typical malformations of the bulldog phenotype. These included the shortened legs, brachygnathia superior, shortening and compressed body, malformed vertebrae, shortening of the vertebral column and palatoschisis [9, 11, 18]. A further missense mutation in *COL2A1* was found in crossbred calves from a Charolais bull and Salers dams with a similar bulldog phenotype [10].

In the present study, we demonstrate a bulldog phenotype in a Miniature Zebu and analyze WGS data of a bulldog calf and its parents in order to identify the potential causal mutation.

## Results

### Phenotype of the affected calf

**Fig. 1:**
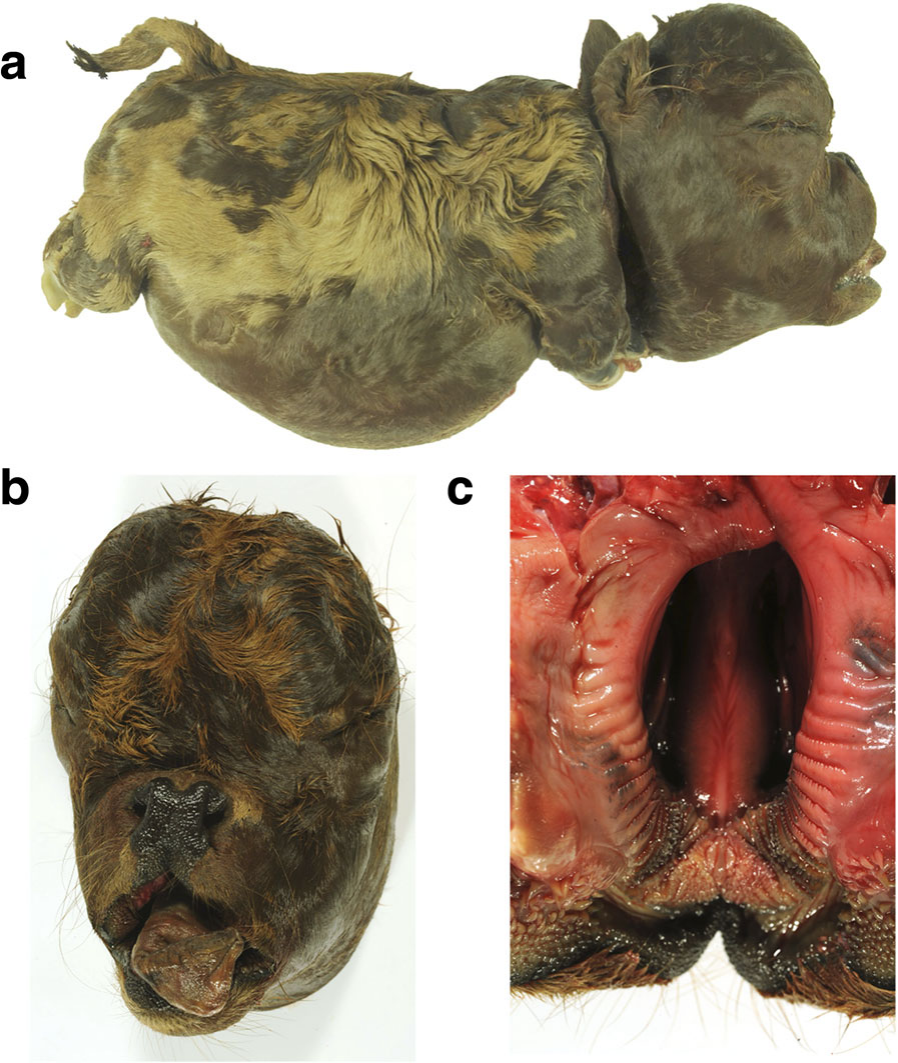
Macroscopical findings. **a** Lateral view of the bulldog Miniature Zebu calf. Note the enlarged and dome-shaped head as well as shortened trunk and limbs. **b** Frontal view of the head. Note the shortened and deformed splanchnocranium with protruded mandible and tongue. **c** Ventral view of the maxilla and hard palate with cleft lip and palate

The head of the affected calf was in relation to its body very large and bulge, whereas the body of the calf appeared short and compressed (Fig. 1a). In addition, the limbs were shortened and stocky, but the phalanges showed normal size. In contrast, the two investigated parents of the affected calf did not show any bone abnormalities. The neck and the spine of the calf were shortened. The buttocks appeared to have an increased muscular mass, which led to dorsocranially-displaced tail. The body showed a fully developed coat, the skin was not wrinkled and abdominal wall was closed. Various craniofacial anomalies were seen including a shortening of the skull base and a bulge forehead (Fig. 1a, b). Furthermore, the calf showed brachygnathia superior, the nose was depressed and the upper jaw shortened in relation to the lower jaw. The bridge of the nose was bent inwards and the upper lip showed a notch (Fig. 1c). In addition, the teeth were spiky, while the prolonged, fleshy tongue protruded from the mouth and was rolled in. The ears were dislocated to a caudal position.

**Fig. 2:**
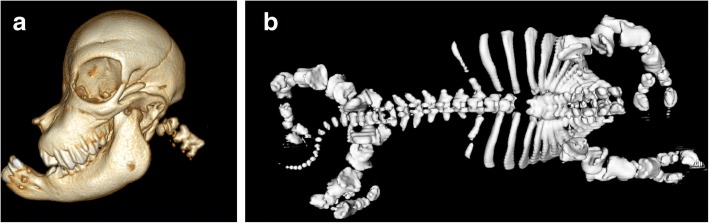
Computer tomography (CT) of skull and body. **a** Computer tomography scan revealed a characteristically shortened nasal region, a bulged forehead, brachygnathia superior and caudodorsally located eye sockets. **b** In the dorsoventral view of the body a significant shortening of the vertebral column was shown. The first ten thoracic vertebrae were fused, the spinous processes were not developed over the entire length and transverse processes of the lumbar spine were only rudimentary. The ribs also showed a shortening, an irregular shape, a variable distance from each other and partial fusion. Severe malformations were also detected in the limbs. Wide distances in-between long bones in the CT scan were a result of impaired mineralization of the epiphyses. In addition, the pelvis and scapula appear small and burly

The CT scan in the lateral view of the head confirmed a shortening of the nose region and a bulging forehead (Fig. 2a). The shape of the forehead was a result of an expansion of the frontal, parietal and occipital bones, which made the neurocranium very prominent and gave the head a spherical appearance. In addition, the head in the lateral view showed brachygnathia superior, prognathism, a notch in the area of the nose and eye sockets that were located caudodorsally. In the craniocaudal view of the skull, a shortening of the ventral nasal conchae and a cleft hard palate was visible. In the dorsoventral view of the body a significant shortening of the vertebral column was found, whereby the number of vertebral bodies was in the normal range with seven cervical vertebrae, thirteen thoracic vertebrae, six lumbar vertebrae, five sacral vertebrae and seven coccygeal vertebrae (Fig. 2b). However, the first ten thoracic vertebrae were fused in the dorsoventral view, and the first ten ribs were partially fused together. The shape of the vertebrae was variable, the spinous processes were not developed over the entire length and transverse processes of the lumbar spine were only rudimentary. A number of the vertebrae bodies had a major failure of the fusion of the right and left dorsal parts. The ribs were shortened, irregularly shaped and had a variable distance from each other. In the latero-lateral view an unusual arrangement of the ribs was obvious. They were arranged rather horizontally instead of vertically. The limbs also showed severe malformations. The long bones were significantly shortened and irregularly shaped. The diaphysis of the long bones were mineralized, but not the epiphyses, which resulted in an extremely wide region of radiopaque material between the individual long bones in CT images. The pelvis was underdeveloped, which made it look small and burly. In addition, the scapula, like the pelvis, showed a developmental delay, which made its appearance small and burly.

### Necropsy

At post mortem examination the bulldog Miniature Zebu calf showed a crown-rump length of 24 cm (9.45 in.) and a body weight of 4.3 kg. The head of the calf was enlarged and dome-shaped with protruded mandible and tongue. A cleft lip and palate was present (Fig. 1c). The splanchnocranium was shortened and deformed. Furthermore, the calf had a shortened vertebral column with reduced sizes of individual vertebrae, a deformed thorax with shortened ribs, as well as shortened and rotated limbs. Findings in other organs included diffuse subcutaneous edema (anasarca) and total lung atelectasis.

### Histopathologic and virological examinantion

Histologically, malformed humerus, femur, tarsal, metatarsal, and metacarpal bones as well as cervical vertebrae showed a disorganization of the physeal cartilage with irregular arrangement of chondrocytes, characteristic of chrondrodysplasia (Additional file 1). The epiphyseal plate was sealed multifocally by deposition of osteoid. In other organs no significant findings were present. By means of cultural examination *bovine virus diarrhoe virus*-infection was excluded.

### Whole genome sequencing and variant detection

In total, 154,417,677 (bulldog calf), 195,556,794 (dam) and 159,320,369 (sire) reads were mapped to the reference genome UMD 3.1. Aligned data had a mean coverage of 8.4X (bulldog calf), 10.7X (dam) and 8.7X (sire) in the bulldog Miniature Zebu family. Based on 33,367,047 called and quality controlled SNPs and 4,415,382 indels, filtering analysis was performed for homozygous mutant genotypes exclusively present in the affected bulldog calf. This analysis resulted in one frameshift and seven missense variants (Additional file 2). After comparison of genes from filtering results with candidate genes known to play a role in chondrodysplasia, dwarfism, growth retardation, proportionate dwarfism and inherited congenital skeletal malformations, only two variants were identified in the candidate gene list located in *ACAN* (*ACAN*:g.20850999insC) and *PKD1* (*PKD1*:g.1643626C > T) (Additional file 3).

### Validation and mutation analysis

**Fig. 3:**
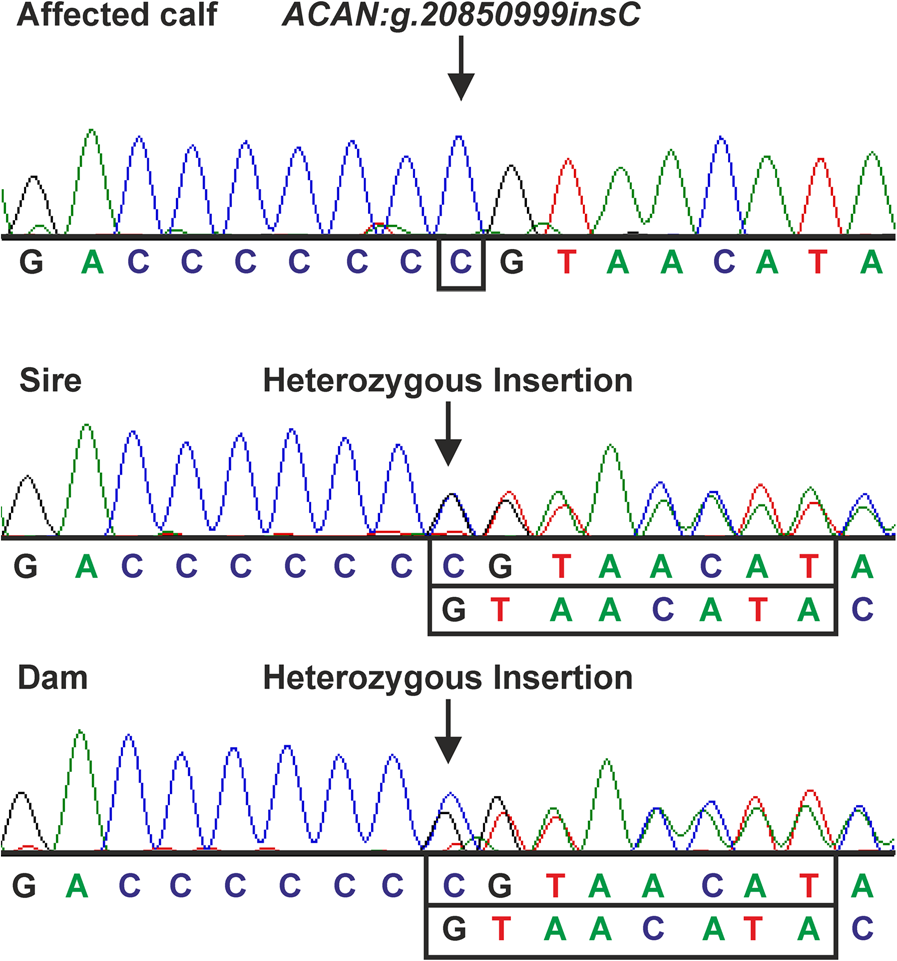
Sequencing analysis of exon 12 of *ACAN.* Sanger sequencing results revealed the homozygous insertion *ACAN*:g.20850999insC in the bulldog calf sample. In its sire and dam the variant was identified in a heterozygous state

Sanger sequencing confirmed the candidate variant *ACAN*:g.20850999insC in exon 12 of *ACAN* (Additional file 4)*.* The affected Miniature Zebu was found to be homozygous for the mutant allele of *ACAN* whereas the dam and sire were heterozygous (Fig. 3). The variant *PKD1*:g.1643626C > T designated as homozygous mutant in WGS data was shown to be heterozygous in the affected calf and its sire and therefore was excluded as candidate variant (Additional file 5).

**Fig. 4:**
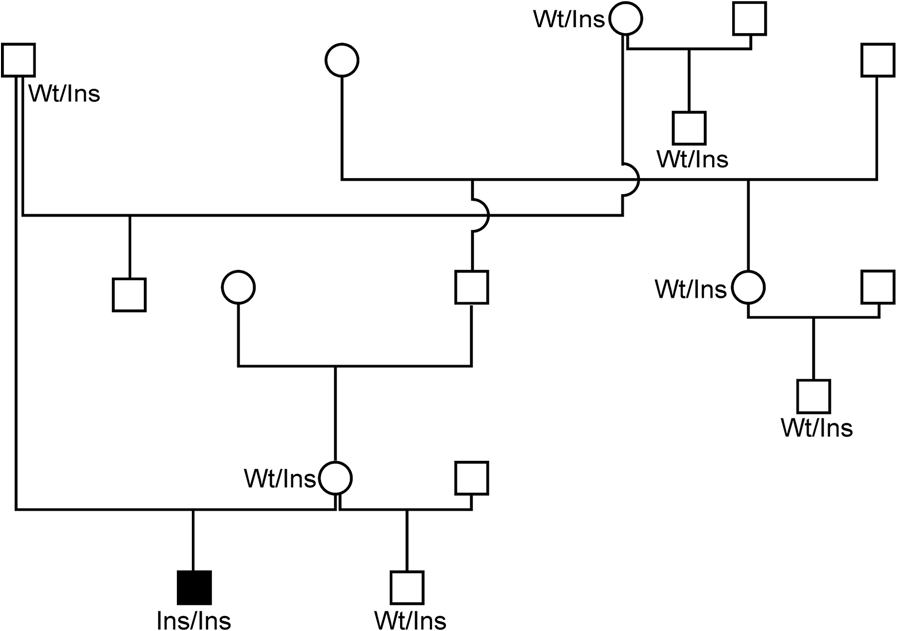
Pedigree of the affected Miniature Zebu phenotypes. In the pedigree of the affected Miniature Zebu, no other animals were affected. Genotypes of *ACAN*:g.20850999insC for investigated individuals are displayed. Filled symbols represent affected individuals and unfilled symbols show unaffected individuals

Further genotyping of the *ACAN* variant in controls showed that the homozygous mutant variant was not present in any other tested individuals (Table 1, Fig. 4). Controls were 88 individuals from eight different taurine cattle breeds, 42 healthy Miniature Zebu not related with members of the herd with the bulldog calf and further five Miniature Zebus from the herd with the proband. The insertion of one base pair (*ACAN*:c.5686insC) is predicted to cause a frameshift (p.Val1898fsTer9) and subsequently a premature stop codon in exon 12. A predicted sequence similarity of 99.53% was found for zebu cattle protein (XP_019838757.1) with cattle protein transcript 202 (ENSBTAP00000021514). Both amino acid (aa) sequences were proposed to be truncated by 423 aa (Additional file 6). Open reading frame finder tool (https://www.ncbi.nlm.nih.gov) suggested the premature stop codon at aa position 1906, whereas wild type ACAN revealed a product size of 2328 aa. This frameshift is predicted to result in a shortened chondroitin sulfate domain and a loss of the C-terminal globular domain 3 (Additional file 7).

**Table 1:** Distribution of the *ACAN*:g.20850999insC variant in 50 Miniature Zebus and 88 individuals from taurine cattle breeds

Breeds	Number of animals	Genotype Ins/Ins	Genotype wt/Ins	Genotype wt/wt
Miniature Zebus				
Affected bulldog calf	1	1	0	0
Obligate carriers	2	0	2	0
Relatives to the affected calf	5	0	5	0
Individuals from other herds	42	0	0	42
Taurine cattle breeds				
Salers	11	0	0	11
Angus	11	0	0	11
Blonde d’Aquitaine	11	0	0	11
German Brown	11	0	0	11
Charolais	11	0	0	11
German Fleckvieh	11	0	0	11
Holstein Friesian	11	0	0	11
Limousin	11	0	0	11

## Discussion

This is the first study describing a novel variant in *ACAN* for chondrodysplasia in Miniature Zebus. Whole genome sequencing data enabled the detection of a homozygous frameshift mutation in an affected calf validated through Sanger sequencing. Thus, validation confirmed NGS data with an average coverage of 8.4X of the affected calf. Even in the first phase of the 1000 bull genomes project with an average coverage of 8.3X a heterozygous causative variant for lethal chondrodysplasia was identified [12]. In addition, the high number of control samples facilitated the exclusion of false positive results for chondrodysplasia-associated variants.

The clinical phenotype of chondrodysplastic Zebu cattle has already been observed in Nganda and Nellore cattle [15, 16]. The clinical findings of these cases were similar to those identified in the Miniature Zebu investigated in this work. Phenotypic characteristics in the affected Miniature Zebu were typical for bulldog syndrome, similar to those previously described in Dexter [1, 3, 7], Holstein Friesian [9, 11, 19], Miniature Scottish Highland [8, 13] and Miniature belted Galloways [8] due to generalized chondrodysplasia. Only two genes, *ACAN* [17] and *COL2A1* [9–12], were so far identified harboring causative variants for these bulldog types. Variants in *ACAN* were detected in exon 1 (BD2) and exon 11 (BD1) in Dexter [17], Miniature Scottish Highland [8, 13] and Miniature Belted Galloway [8]. The BD variants lead to different shortenings of a keratan sulfate domain. BD1 leads to a complete loss of the chondroitin sulfate domain and the globular domain, whereas BD2 causes a shift of the open reading frame and produces a shortened protein of unknown function [17]. It is a striking feature that all these bulldog calves caused by mutant *ACAN* variants were from miniature cattle breeds. These results suggest that bulldog syndromes associated with *ACAN* variants are specific for miniature cattle breeds. Aggrecan proteoglycan is an important component of the extracellular matrix in the articular cartilage [20]. ACAN which is located in the extracellular matrix can form a hydrated gel affecting chondrocyte-chondrocyte and chondrocyte-matrix interactions [20]. Proteoglycan mutation were proposed to disturb chondrocyte column formation and demarcation of proliferative and hypertrophic zones of epiphyseal cartilage [21]. This results in defects of differentiation and maintenance of the skeletal elements [22]. Due to the fact that ACAN is the primary proteoglycan component of the extracellular matrix of cartilage growth plate and other cartilaginous tissues [23], *ACAN* variants were also found to be associated with genetic skeletal diseases in other species like chicken [24, 25], mouse [26, 27], human [6] and horse [28]. A characteristic finding in humans was a short stature. The seven known variants in *ACAN* [6, 23, 29–32], which are responsible for this short stature in human are in different exons, but there are two mutations in exon 12. All variants have in common that they produce a truncated protein due to a premature stop codon. In three variants, the premature stop codon was located in the C-type lectin domain located within the G3 domain [23, 30, 31]. In other cases, the stop codon was located proximal to the G1 and G2 [23], CS-1 [6] and CS-2 domain [29]. Aggrecanopathies in other mammalian species have been shown to be perinatally or embryonically lethal in a homozygous state [17, 26, 28, 33, 34]. Furthermore, all affected individuals had short limbs and were thus, affected by disproportionate dwarfism [26, 28, 33–35]. The histological changes seen in the shortened legs of affected calves are due to a disturbance of endochondral ossification [7].

Disturbances of the endochondral ossification caused by *ACAN* mutations may lead to genetic skeletal diseases [36]. In animals, different exons of *ACAN* are affected by *ACAN* mutations [26, 34]. However, all variants of *ACAN* lead to shortened proteins due to a premature stop codon at different positions. An *ACAN* variant in the horse was found to affect the C-lectin domain [28]. However, it can be assumed that this, as in humans, is analogous to the G3 domain [23]. The truncated protein in nanomelia is retained and accumulated in small cytoplasmic structures corresponding to extensions of the endoplasmic reticulum (ER) [37]. For this reason, chondrocytes of chicken affected by nanomelia do not contain the mutant aggrecan protein in cartilage extracellular matrix (ECM). In the Cmd-affected mouse, the truncated aggrecan protein was not detected in the endoplasmic reticulum and was consequently missing in the ECM [38]. These findings demonstrated that the reduction or absence of ACAN in the ECM is causing the chondrodysplasia phenotype [6]. Further studies are required to establish whether the truncated protein is synthesized and has an effect on other proteins within the endoplasmic reticulum and on chondrocyte function. Based on the results in mouse and human and histopathological findings in the present bulldog case, we suppose that the potential causal mutation in *ACAN* identified in this study on a bulldog Miniature Zebu leads to a truncated transcript and the resulting truncated protein might accumulate in the endoplasmic reticulum causing severe generalized bone malformations.

## Conclusions

In the present study, we found a novel mutation in *ACAN* resulting in a recessive lethal chondrodysplasia in Miniature Zebu cattle. This is the first study on *Bos indicus*, which successfully used whole genome sequencing for the identification of a causal chondrodysplasia variant. A diagnostic test for this mutation is now available for Miniature Zebu breeders preventing further cases of bulldog in Miniature Zebu by targeted matings.

## Methods

### Animals

In total, 50 samples from privately owned Miniature Zebu samples were collected at five farms performing non-commercial breeding in Germany. The samples per herd comprised 3, 8, 10, 13 and 16 animals. The genomic DNA of 50 Miniature Zebu and further 88 individuals from different European cattle breeds as controls was isolated from EDTA-blood, hair root or tissue samples. In total eight Miniature Zebus (parents, affected calf and five relatives) were sampled from the herd where the affected Miniature Zebu was born. The other 42 samples of Miniature Zebus came from herds where no cases of bulldog calves have been observed to date. The 88 samples from other breeds included Salers, Angus, Blonde d’Aquitaine, Brown Swiss, Charolais, Holstein Friesian, Fleckvieh and Limousin. Veterinarians under avoiding any unnecessary pain to animals did sampling in this investigation. During this experiment, no animals died or had to be euthanized.

### Clinical examination

A Miniature Zebu cow has given birth to a male calf after a gestation length of 7 months. At birth, this male calf was already dead. Due to a severe malformed head, a fetotomy had to be performed by the veterinary practitioner. The head was dissected from the body in order to save the life of the dam. The head and the body of the bulldog Miniature Zebu underwent a computed tomography (CT) scan in sternal recumbency using a multislice helical CT scanner (Brilliance-™-CT BigBore Oncology, Philips Medical Systems, Best, The Netherlands). A slice thickness for the head of 1 mm and settings of 120 kV/ 300 mAs and for the body a slice thickness of 2 mm and settings of 140 kV/ 300 mAs were set for analysis. A 512 × 512 matrix was used.

### Histopathological examination

A full necropsy of the bulldog Miniature Zebu calf was carried out. Samples from long bones (humerus, radius, metatarsal, metacarpal bones), including articular cartilage, epiphysis, epiphyseal plate, metaphysis and diaphysis, as well as tarsus and cervical vertebrae were fixed in 10% formalin for 48 h, followed by decalcification in HNO_3_ solution (5%) for 24 h. Afterwards, tissue samples were embedded in paraffin and 4 μm thick paraffin sections were cut and stained with hematoxylin and eosin (HE) for histological examination. Photographs were taken with a light microscope (Olympus BX51, Olympus, Hamburg, Germany) with an Olympus camera DP72 and Olympus cellSense software.

### Whole-genome sequencing

To identify a potential causal variant for bulldog in Miniature Zebu, genomic DNA from EDTA-blood samples of the sire and the dam and tissue from the affected calf was extracted using a standard salting out procedure with chloroform. Libraries were prepared and quality controlled according to standard protocols using the NEBNext Ultra DNA Library Prep Kit for Illumina (New England BioLabs, Ipswich, MA, USA). Libraries were run on an Illumina NextSeq500 in a 2 × 150 bp in paired-end mode. Quality control was performed using fastqc 0.11.5 [39] and reads were trimmed using PRINSEQ (V 0.20.4) [40]. WGS data of the bulldog calf, sire and dam were deposited in NCBI Sequence Read Archive (http://www.ncbi.nlm.nih.gov/sra) under SRA accession number SRP132671 (PRJNA433787). After quality control mapping was performed to the bovine reference genome UMD 3.1. (Ensembl) using BWA 0.7.13 [41]. Bam-files were sorted and indexed using SAMtools 1.3.1 [42] and duplicates were marked using Picard tools (http://broadinstitute.github.io/picard/, version 2.9.4). In a further step, GATK version 4.0 [43] including Base Quality Score Recalibration (BQSR), Haplotype Caller and Variant Recalibrator were used for variant calling. These data of the affected calf, dam and sire were compared with WGS data of 51 different individuals from the breeds Holstein, German Brown, Charolais, Hereford, Limousin, Tyrolean Grey and Vorderwald [44]. For further analyses, variants with a read depth of 2–999 and quality score values > 20 were selected. We filtered out variants, which were homozygous mutant for the bulldog Miniature Zebu, but not homozygous mutant in the sire and dam and homozygous wild type in all other animals using SAS, version 9.4 (Statistical Analysis System, Cary, NC, USA). Variants flagged with annotation accuracy warnings were omitted. Finally, only those variants with high or moderate effects according to prediction toolbox SNPEff version 4.3 q (2017-08-30, SNPEff database UMD3.1.86) [45] were chosen for further inspection. Variant effect prediction was performed using the Variant Effect Predictor [46] based on UMD 3.1 assembly for SIFT [47] predictions. A candidate gene list for the terms chondrodysplasia, dwarfism, growth retardation, proportionate dwarfism and inherited congenital skeletal malformations was prepared using NCBI Gene database and supplemented with further genes identified in previous studies [9, 11].

### Validation and mutation analysis

For validation of detected mutant candidate variants, Sanger sequencing was performed for exon 12 of *ACAN* and for exon 15 in *PKD1* in the affected Miniature Zebu, dam, sire and three further individuals. A sample of muscle tissue from the affected calf, EDTA-blood from sire and dam as well as hair samples from the other three individuals from the affected herd were available for DNA isolation. For DNA isolation a standard saline precipitation method was performed [48]. Primer3 tool (version 0.4.0, http://bioinfo.ut.ee/primer3-0.4.0/) was applied for primer design (Additional file 8). For DNA derived from hair samples, a second primer pair was used for an additional PCR on the already obtained PCR-product to increase the specificity of reactions. PCRs were performed in 22-μl reaction volumes containing 2 μl DNA, 1.5 mM deoxyribonucleoside triphosphates, 5 pmol of each primer, 1.5 U of *Taq* polymerase in the reaction buffer supplied by the manufacturer (MP Biomedicals, Eschwege, Germany) and with enhancer reagent (Qiagen, Hilden, Germany) for optimizing reaction conditions. After a 5 min initial denaturation at 95 °C, 42 cycles of 30 s at 94 °C, 30 s at 58 °C, and 45 s at 72 °C was run on a Thermocycler TProfessional 96 (Biometra, Göttingen, Germany). PCR products were sequenced on Applied Biosystems 3500 Series Genetic Analyzer (Thermo Fisher Scientific, Waltham, MA, USA). Sequence data were analysed using Sequencher 4.8 (Genes Codes, Ann Arbor, MI, USA).

In addition, the Miniature Zebu bulldog associated variant in exon 12 of *ACAN* was genotyped in the affected calf, sire, dam, five other individuals of the affected herd and further 42 healthy Miniature Zebu and 88 individuals from taurine breeds including Salers, Angus, Blonde d’Aquitaine, Braunvieh, Charolais, Holstein Friesian, Fleckvieh, Limousin using a Kompetitive Allele Specific PCR (KASP) assay (LGC Genomics, Middlesex, UK; He et al., 2014). KASP reaction was performed using 5 μl KASP Master Mix 2× (LGC Genomics), 0.14 μl KASP Assay mix (two allele-specific primers, one common primer designed by LGC) and 5 μl template DNA with a concentration of 10 ng/μl and run on an ABI7300 real-time system for 96 well plates (Additional file 9).

In addition, *ACAN* transcripts found in zebu and cattle were investigated for predicted open reading frames using ORFfinder (NCBI, https://www.ncbi.nlm.nih.gov) and compared with each other using Clustal Omega [49]. Predicted protein domains were identified using UniProdKB database (P13608_BOVIN) [50] and data from a previous study [17]. The variant *ACAN*:g.20850999insC was submitted to European Variant Archive (PRJEB24930).

## Additional files

Additional file 1: Histopathological findings of the epiphyseal plate. Note undulated epiphyseal plate with irregular arrangement of chondrocytes and osteoid deposition (asterisks). Inset: higher magnification. HE, 40×. (TIF 2137 kb)

Additional file 2: Results from the filtering analysis for exclusively homozygous variants in the affected Miniature Zebu calf. Potential chondrodysplasia-associated genes are given in bold. Bovine chromosome, position, mutation, amino acid change, consequence, genotypes of the affected calf, sire and dam, gene, transcript and SIFT predictions are shown. (DOCX 17 kb)

Additional file 3: Candidate gene list analysis. Genes potentially involved in chondrodysplasia, dwarfism, growth retardation, proportionate dwarfism and inherited congenital skeletal malformations in mammals were identified using NCBI Gene database and gene lists from previous studies [9, 11, 15]. The respective bovine chromosome, position, genes, gene ID and name are shown. Genes identified in filtering analysis in the present case of a dwarf Miniature Zebu are printed in bold. (DOCX 29 kb)

Additional file 4: Gene model of the candidate gene *ACAN* of *Bos indicus* and *Bos taurus*. The transcript of *ACAN* of *Bos indicus* harbors sixteen exons (a) and the transcript *ACAN*-202 of *Bos taurus* seventeen exons (b) according to NCBI database. However, the transcripts of *Bos indicus* and *Bos taurus* are identical at 99.53%. The candidate variant *ACAN*:g.20850999insC identified in this analysis and further two variants BD1 and BD2 found in a previous study are shown. Black boxes indicate consecutively numbered exons. White areas in the black boxes show the untranslated region. (TIF 253 kb)

Additional file 5: Sequencing analysis of *PKD1* variant. Sanger sequencing results revealed the variant *PKD1*:g.1643626C > T heterozygous in the bulldog calf and its sire. (TIF 675 kb)

Additional file 6: Variant effect on protein sequence. Amino acid sequence of Zebu cattle transcript (*Bos indicus*, XP_019838757.1) and the analogous protein sequence of *Bos taurus* transcript 202 (ENSBTAP00000021514) are shown. The insertion *ACAN*:g.20850999insC is predicted to cause a modified amino acid sequence at position 1898 (p.Val1898fsTer9) and a premature stop codon after nine amino acids. Asterisks represent identical amino acids and periods display similar amino acids. (TIF 492 kb)

Additional file 7: Predicted protein domains. The protein model of *ACAN* is predicted to contain a signal preptide (1–16 aa) as well as two globular domains (G1 and G2) in the C-terminal region enclosing an interglobular region (IGD), followed by a keratin sulfate domain (KS), chondroitin sulfate domain (CS-2, 1433–2112 aa) and a C-terminal globular region (G3) (a). The variant p.Val1898ArgfsTer9 in the affected calf is predicted to result in a truncation of the CS-2 domain and subsequently a loss of C-terminal G3 (b). The arrow shows the amino acid substitution of valine to arginine which results subsequently in a frameshift and a termination codon after nine further amino acids. (TIFF 140 kb)

Additional file 8: Primer pairs used for Sanger sequencing. Forward and reverse primers for validation of candidate variants found in *ACAN* and *PKD1*, amplicon size (AS), annealing temperature (AT) and number of cycles are shown. DNA derived from hair samples required an internal primer (*ACAN*_2). (DOCX 13 kb)

Additional file 9: Primer pairs used for genotyping the *ACAN*:g.20850999insC variant. The single nucleotide candidate variant located in *ACAN* was genotyped using a Kompetitive Allele Specific PCR (KASP) assay. Forward and reverse primers, fluorescent labels (FAM/VIC), annealing temperature (AT) and number of cycles are shown. (DOCX 13 kb)

## Abbreviations

aa: Amino acid
*ACAN*: *Aggrecan*
BD1: Bulldog dwarfism 1
BD2: Bulldog dwarfism 2
BTA: *Bos taurus autosome*
cmd: Cartilage matrix deficiency
*COL2A1*: *Collagen type II alpha 1 chain*
CS-1: Chondroitin sulfate domain 1
CS-2: Chondroitin sulfate domain 2
CT: Computed tomography
ECM: Extracellular matrix
EDTA: Ethylenediaminetetraacetic acid
ER: Endoplasmic reticulum
G1: Globular region 1
G2: Globular region 2
G3: Globular region 3
HE: Hematoxylin and eosin
KASP: Kompetitive Allele Specific PCR
*PKD1*: *Polycystin 1, transient receptor potential channel interacting*
SRA: Sequence Read Archive
Val: Valin
WGS: Whole genome sequencing

## References

[1] Seligmann C (1904). Cretinism in calves. J Pathol.

[2] Huston K, Saperstein G, Steffen D, Millar P, Lauvergne J (2000). 2. Clinical, pathological and other visible traits loci except coat colour (category 2). Publication-European Association for Animal Production.

[3] Crew F (1924). The bull-dog calf: a contribution to the study of achondroplasia. SAGE publications.

[4] Dirksen G (2006). Innere Medizin und Chirurgie des Rindes. Vol. 9.10.7.2.

[5] Maxie MG (2007). Jubb, Kennedy, and Palmer's pathology of domestic animals.

[6] Gleghorn L, Ramesar R, Beighton P, Wallis G (2005). A mutation in the variable repeat region of the aggrecan gene (AGC1) causes a form of spondyloepiphyseal dysplasia associated with severe, premature osteoarthritis. Am J Hum Genet.

[7] Harper P, Latter M, Nicholas F, Cook R, Gill P (1998). Chondrodysplasia in Australian Dexter cattle. Aust Vet J.

[8] Dittmer K, Thompson K, Hogan T. Severe generalised chondrodysplasia in miniature cattle breeds. N Z Vet J. 2017;(just-accepted):1–7. 10.1080/00480169.2017.1329036 28504055

[9] Agerholm JS, Menzi F, McEvoy FJ, Jagannathan V, Drögemüller C (2016). Lethal chondrodysplasia in a family of Holstein cattle is associated with a de novo splice site variant of COL2A1. BMC Vet Res.

[10] Bourneuf E, Otz P, Pausch H, Jagannathan V, Michot P, Grohs C, Piton G, Ammermüller S, Deloche M-C, Fritz S. Rapid discovery of De novo deleterious mutations in cattle enhances the value of livestock as model species. Sci Rep. 2017;7. 10.1038/s41598-017-11523-3 PMC5597596 28904385

[11] Reinartz S, Mohwinkel H, Sürie C, Hellige M, Feige K, Eikelberg D, Beineke A, Metzger J, Distl O (2017). Germline mutation within COL2A1 associated with lethal chondrodysplasia in a polled Holstein family. BMC Genomics.

[12] Daetwyler Hans D, Capitan Aurélien, Pausch Hubert, Stothard Paul, van Binsbergen Rianne, Brøndum Rasmus F, Liao Xiaoping, Djari Anis, Rodriguez Sabrina C, Grohs Cécile, Esquerré Diane, Bouchez Olivier, Rossignol Marie-Noëlle, Klopp Christophe, Rocha Dominique, Fritz Sébastien, Eggen André, Bowman Phil J, Coote David, Chamberlain Amanda J, Anderson Charlotte, VanTassell Curt P, Hulsegge Ina, Goddard Mike E, Guldbrandtsen Bernt, Lund Mogens S, Veerkamp Roel F, Boichard Didier A, Fries Ruedi, Hayes Ben J (2014). Whole-genome sequencing of 234 bulls facilitates mapping of monogenic and complex traits in cattle. Nature Genetics.

[13] Cabrera LC, McNabb BR, Woods SE, Cartoceti AN, Busch RC (2016). Hydrops associated with chondrodysplasia of the fetus in a miniature Scottish Highland cow. J Am Vet Med Assoc.

[14] Dittmer K, Hogan T, Thompson K (2013). A case of bulldog-type chondrodysplasia in a miniature Scottish highland calf. J Comp Pathol.

[15] Moura E, Prado A, Pimpão C, Murakami C, Ribeiro D (2014). Genetic and Pathoanatomical features of the bovine prenatal lethal chondrodysplasia. Hereditary Genet.

[16] Carmichael J (1933). Bulldog calf in African cattle. Nature.

[17] Cavanagh JA, Tammen I, Windsor PA, Bateman JF, Savarirayan R, Nicholas FW, Raadsma HW (2007). Bulldog dwarfism in Dexter cattle is caused by mutations in ACAN. Mamm Genome.

[18] Agerholm JS, Arnbjerg J, Andersen O (2004). Familial chondrodysplasia in Holstein calves. J Vet Diagn Investig.

[19] Naito K, Maruyama M, Dobashi K, Tanimura N, Kimura K, Haritani M, Nakajima Y (2002). Congenital chondrodysplastic dwarfism with dyshematopoiesis in a Holstein calf. J Vet Med Sci.

[20] Yang BB, Zhang Y, Cao L, Yang BL (1998). Aggrecan and link protein affect cell adhesion to culture plates and to type II collagen. Matrix Biol.

[21] Bingel S, Sande R (1982). Chondrodysplasia in the Norwegian elkhound. Am J Pathol.

[22] Schwartz NB, Domowicz M (2002). Chondrodysplasias due to proteoglycan defects. Glycobiology.

[23] Nilsson O, Guo MH, Dunbar N, Popovic J, Flynn D, Jacobsen C, Lui JC, Hirschhorn JN, Baron J, Dauber A (2014). Short stature, accelerated bone maturation, and early growth cessation due to heterozygous aggrecan mutations. J Clin Endocrinol Metab.

[24] Velleman S (2000). The role of the extracellular matrix in skeletal development. Poult Sci.

[25] Vertel BM, Walters LM, Grier B, Maine N, Goetinck PF (1993). Nanomelic chondrocytes synthesize, but fail to translocate, a truncated aggrecan precursor. J Cell Sci.

[26] Watanabe H, Kimata K, Line S, Strong D (1994). Gao L-y, Kozak CA, Yamada Y: mouse cartilage matrix deficiency (cmd) caused by a 7 bp deletion in the aggrecan gene. Nat Genet.

[27] Krueger RC, Kurima K, Schwartz NB (1999). Completion of the mouse aggrecan gene structure and identification of the defect in the cmd-Bc mouse as a near complete deletion of the murine aggrecan gene. Mamm Genome.

[28] Metzger J, Gast AC, Schrimpf R, Rau J, Eikelberg D, Beineke A, Hellige M, Distl O (2017). Whole-genome sequencing reveals a potential causal mutation for dwarfism in the miniature Shetland pony. Mamm Genome.

[29] Quintos JB, Guo MH, Dauber A (2015). Idiopathic short stature due to novel heterozygous mutation of the aggrecan gene. J Pediatr Endocrinol Metab.

[30] Tompson SW, Merriman B, Funari VA, Fresquet M, Lachman RS, Rimoin DL, Nelson SF, Briggs MD, Cohn DH, Krakow D (2009). A recessive skeletal dysplasia, SEMD aggrecan type, results from a missense mutation affecting the C-type lectin domain of aggrecan. Am J Hum Genet.

[31] Stattin E-L, Wiklund F, Lindblom K, Önnerfjord P, Jonsson B-A, Tegner Y, Sasaki T, Struglics A, Lohmander S, Dahl N (2010). A missense mutation in the aggrecan C-type lectin domain disrupts extracellular matrix interactions and causes dominant familial osteochondritis dissecans. Am J Hum Genet.

[32] Stattin E-L, Tegner Y, Domellöf M, Dahl N (2008). Familial osteochondritis dissecans associated with early osteoarthritis and disproportionate short stature. Osteoarthr Cartil.

[33] Landauer W (1965). Nanomelia, a lethal mutation of the fowl. J Hered.

[34] Bell L, Juriloff D, Harris M (1986). A new mutation at the cmd locus in the mouse. J Hered.

[35] Wai AW, Ng LJ, Watanabe H, Yamada Y, Tam PP, Cheah KS (1998). Disrupted expression of matrix genes in the growth plate of the mouse cartilage matrix deficiency (cmd) mutant. genesis.

[36] Gibson BG, Briggs MD (2016). The aggrecanopathies; an evolving phenotypic spectrum of human genetic skeletal diseases. Orphanet J Rare Dis.

[37] Vertel BM (1995). The ins and outs of aggrecan. Trends Cell Biol.

[38] Watanabe H, Nakata K, Kimata K, Nakanishi I, Yamada Y (1997). Dwarfism and age-associated spinal degeneration of heterozygote cmd mice defective in aggrecan. Proc Natl Acad Sci.

[39] Andrews S: FastQC: A quality control tool for high throughput sequence data. In: *Reference Source.* http://www.bioinformatics.babraham.ac.uk/projects/fastqc/; 2010.

[40] Schmieder R, Edwards R (2011). Quality control and preprocessing of metagenomic datasets. Bioinformatics.

[41] Li H, Durbin R (2009). Fast and accurate short read alignment with burrows–wheeler transform. Bioinformatics.

[42] Li H, Handsaker B, Wysoker A, Fennell T, Ruan J, Homer N, Marth G, Abecasis G, Durbin R (2009). Genome project data processing S: the sequence alignment/map format and SAMtools. Bioinformatics.

[43] McKenna A, Hanna M, Banks E, Sivachenko A, Cibulskis K, Kernytsky A, Garimella K, Altshuler D, Gabriel S, Daly M (2010). The genome analysis toolkit: a MapReduce framework for analyzing next-generation DNA sequencing data. Genome Res.

[44] Braun M, Reinartz S, Heppelmann M, Rehage J, Sürie C, Distl O, Metzger J (2018). Curly coat caused by a keratin 27 variant was transmitted from Fleckvieh into German Angus. Anim Genet.

[45] Cingolani P, Platts A, Wang le L, Coon M, Nguyen T, Wang L, Land SJ, Lu X, Ruden DM (2012). A program for annotating and predicting the effects of single nucleotide polymorphisms, SnpEff: SNPs in the genome of Drosophila melanogaster strain w1118; iso-2; iso-3. Fly (Austin).

[46] McLaren W, Pritchard B, Rios D, Chen Y, Flicek P, Cunningham F (2010). Deriving the consequences of genomic variants with the Ensembl API and SNP effect predictor. Bioinformatics.

[47] Kumar P, Henikoff S, Ng PC (2009). Predicting the effects of coding non-synonymous variants on protein function using the SIFT algorithm. Nat Protoc.

[48] Miller S, Dykes D, Polesky H (1988). A simple salting out procedure for extracting DNA from human nucleated cells. Nucleic Acids Res.

[49] Sievers F, Wilm A, Dineen D, Gibson TJ, Karplus K, Li W, Lopez R, McWilliam H, Remmert M, Söding J (2011). Fast, scalable generation of high-quality protein multiple sequence alignments using Clustal omega. Mol Syst Biol.

[50] Consortium U (2016). UniProt: the universal protein knowledgebase. Nucleic Acids Res.

