# Supplementary figures and images for: A recessive lethal chondrodysplasia in a miniature zebu family results from an insertion affecting the chondroitin sulfat domain of aggrecan
Source: BMC Genet. 2018 Oct 11;19:91. doi: 10.1186/s12863-018-0678-8 (PMC6180608; doi:10.1186/s12863-018-0678-8)

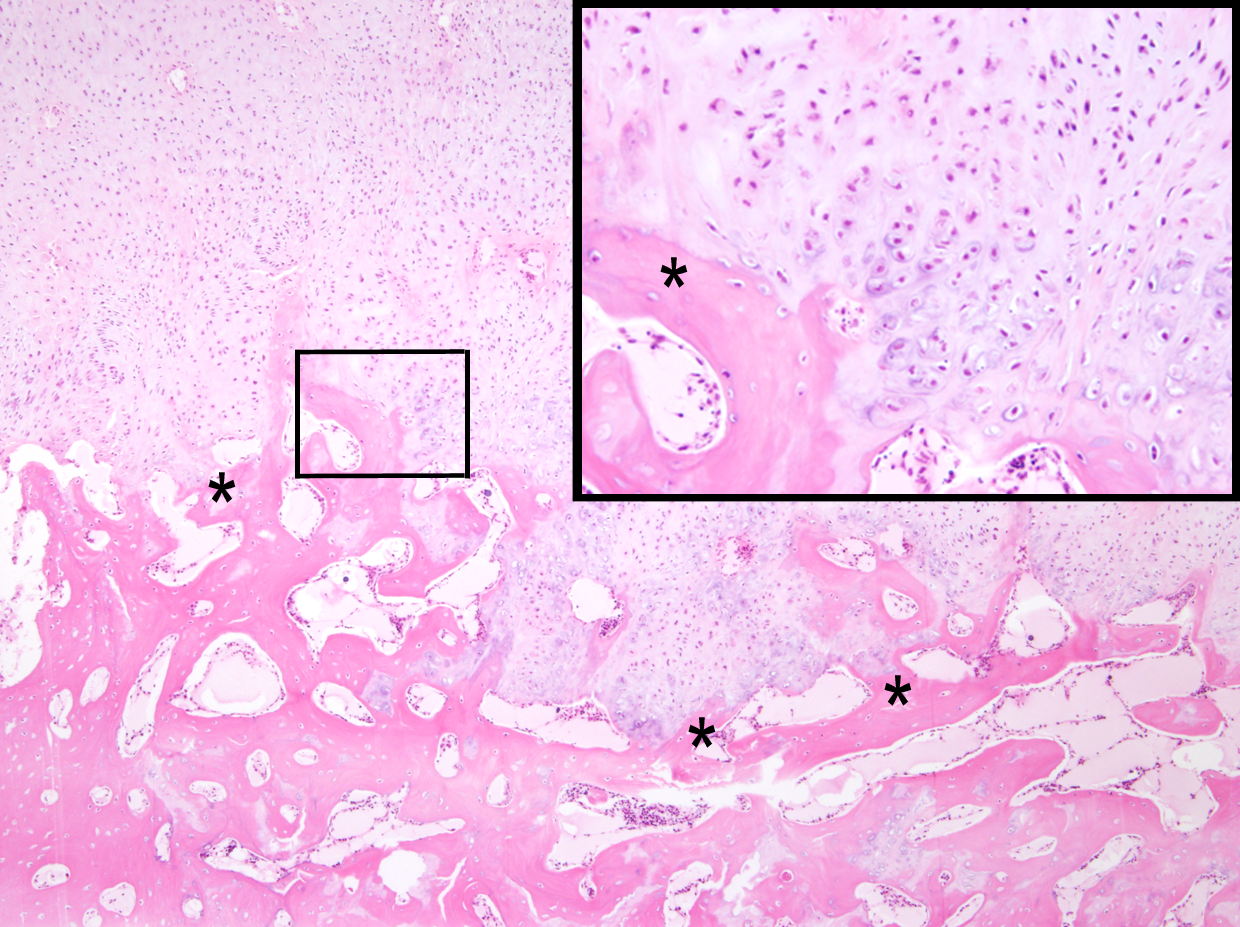

Supplement: Supplementary file 1 — Histopathological findings of the epiphyseal plate. Note undulated epiphyseal plate with irregular arrangement of chondrocytes and osteoid deposition (asterisks). Inset: higher magnification. HE, 40×. (TIF 2137 kb) [file 12863_2018_678_MOESM1_ESM.tif]

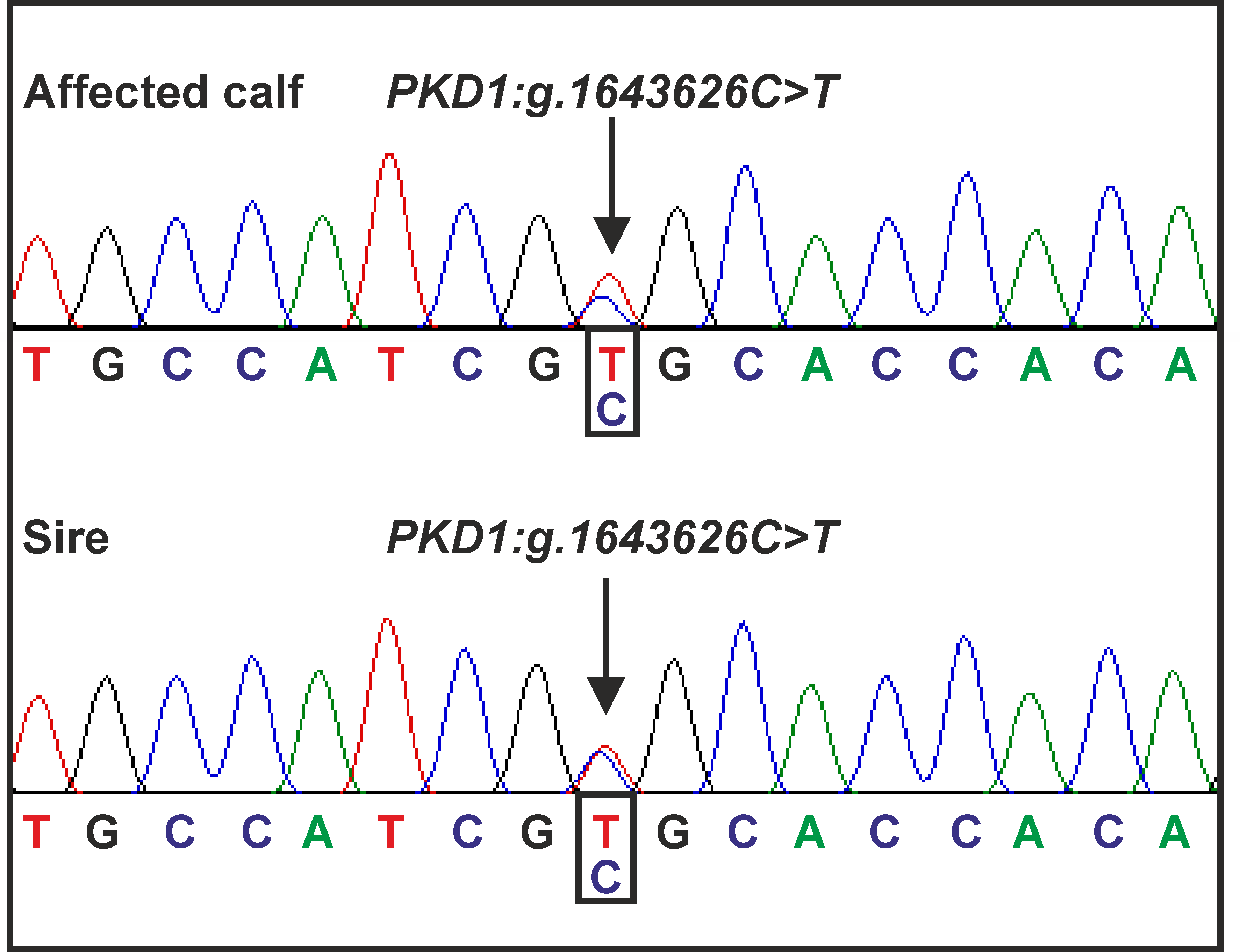

Supplement: Supplementary file 5 — Sequencing analysis of PKD1 variant. Sanger sequencing results revealed the variant PKD1:g.1643626C > T heterozygous in the bulldog calf and its sire. (TIF 675 kb) [file 12863_2018_678_MOESM5_ESM.tif]

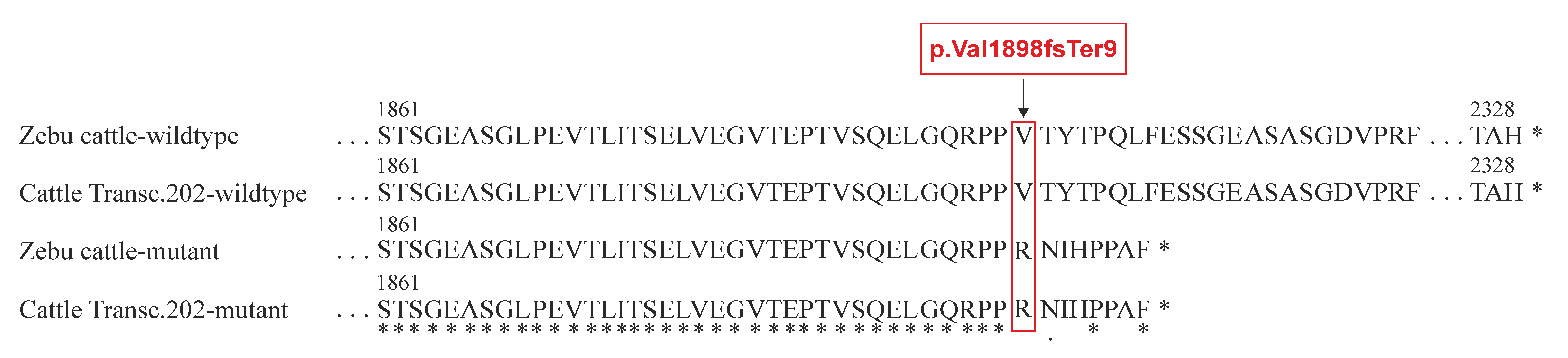

Supplement: Supplementary file 6 — Variant effect on protein sequence. Amino acid sequence of Zebu cattle transcript (Bos indicus, XP_019838757.1) and the analogous protein sequence of Bos taurus transcript 202 (ENSBTAP00000021514) are shown. The insertion ACAN:g.20850999insC is predicted to cause a modified amino acid sequence at position 1898 (p.Val1898fsTer9) and a premature stop codon after nine amino acids. Asterisks represent identical amino acids and periods display similar amino acids. (TIF 492 kb) [file 12863_2018_678_MOESM6_ESM.tif]
