# Supplementary material for: A recessive lethal chondrodysplasia in a miniature zebu family results from an insertion affecting the chondroitin sulfat domain of aggrecan
Source: BMC Genet. 2018 Oct 11;19:91. doi: 10.1186/s12863-018-0678-8 (PMC6180608; doi:10.1186/s12863-018-0678-8)
Supplement: Supplementary file 2 — Results from the filtering analysis for exclusively homozygous variants in the affected Miniature Zebu calf. Potential chondrodysplasia-associated genes are given in bold. Bovine chromosome, position, mutation, amino acid change, consequence, genotypes of the affected calf, sire and dam, gene, transcript and SIFT predictions are shown. (DOCX 17 kb) [file 12863_2018_678_MOESM2_ESM.docx]

**Additional file 2** Results from filtering analysis for exclusively homozygous variants in the affected Miniature Zebu calf. Potential chondrodysplasia-associated genes are given in bold. Bovine chromosome, position, mutation, amino acid change, consequence, gene, transcript and SIFT predictions as well as genotypes of the affected calf, sire and dam are shown.

| Bovine chromosome | Position | Mutation | Amino acid change | Concequence | Genotype bulldog calf | Genotype  dam | Genotype  sire | Gene (transcript) | SIFT |
| --- | --- | --- | --- | --- | --- | --- | --- | --- | --- |
| 7 | 33001966 | T>C | Asp>Gly | missense variant | 1/1 | 0/0 | 0/1 | ENSBTAG00000019881 (ENSBTAT00000056075) | tolerated (0.22) |
| 7 | 55041018 | G>A | Thr>Met | missense variant | 1/1 | 0/1 | 0/1 | ENSBTAG00000045651 (ENSBTAT00000064077) | tolerated (62.00) |
| 11 | 44780354 | G>C | Pro>Ala | missense variant | 1/1 | 0/1 | 0/1 | SULT1C3 (ENSBTAT00000003630) | tolerated (0.09) |
| 15 | 80991272 | G>A | Arg>Cys | missense variant | 1/1 | 0/1 | 0/1 | OR5R1 (ENSBTAT00000065250) | tolerated (0.11) |
| 21 | 20850998 | A>AC | Val>fs/Val>fs | frameshift variant | 1/1 | 0/1 | 0/1 | **ACAN** (ENSBTAT00000021512/  ENSBTAT00000021514) | NA/NA |
| 21 | 71469734 | C>T | Glu>Lys | missense variant | 1/1 | 0/1 | 0/0 | IGHE (ENSBTAT00000065204) | tolerated (0.83) |
| 25 | 1643626 | C>T | Arg>His | missense variant | 1/1 | 0/0 | 0/1 | **PKD1** (ENSBTAT00000027480) | deleterious (0.00) |
| 30 | 56638479 | G>A | Glu>Lys | missense variant | 1/1 | 0/1 | 0/0 | ENSBTAG00000045706 (ENSBTAT00000064519) | NA |
|  |  |  |  |  |  |  |  |  |  |
