# Supplementary material for: A recessive lethal chondrodysplasia in a miniature zebu family results from an insertion affecting the chondroitin sulfat domain of aggrecan
Source: BMC Genet. 2018 Oct 11;19:91. doi: 10.1186/s12863-018-0678-8 (PMC6180608; doi:10.1186/s12863-018-0678-8)
Supplement: Supplementary file 3 — Candidate gene list analysis. Genes potentially involved in chondrodysplasia, dwarfism, growth retardation, proportionate dwarfism and inherited congenital skeletal malformations in mammals were identified using NCBI Gene database and gene lists from previous studies [9, 11, 15]. The respective bovine chromosome, position, genes, gene ID and name are shown. Genes identified in filtering analysis in the present case of a dwarf Miniature Zebu are printed in bold. (DOCX 29 kb) [file 12863_2018_678_MOESM3_ESM.docx]

**Additional file 3** Candidate gene list analysis. Genes potentially involved in chondrodysplasia, dwarfism, growth retardation, proportionate dwarfism and inherited congenital skeletal malformations in mammals were identified using NCBI Gene database and gene lists from previous studies (Agerholm et al. 2016; Moura et al. 2014; Reinartz et al. 2017). The bovine chromosome, position, genes, gene ID and name are shown. Genes identified in filtering analysis in the dwarf Miniature Zebu are printed in bold.

| Bovine  chromosome | Position in bp  (UMD3.1 assembly) | Bovine gene | Gene ID | Gene name |
| --- | --- | --- | --- | --- |
| 1 | 97220035-97252900 | *SLC2A2* | ENSBTAG00000005386 | solute carrier family 2 member 2 |
| 2 | 19510421-19510691 | *AGPS* | ENSBTAG00000009897 | alkylglycerone phosphate synthase |
| 2 | 131478258-131588307 | *HSPG2* | ENSBTAG00000017122 | heparan sulfate proteoglycan 2 |
| 2 | 120392626-120397268 | *NPPC* | ENSBTAG00000003253 | natriuretic peptide C |
| 2 | 104975964-104987392 | *SHOX* | ENSBTAG00000026788 | short stature homeobox |
| 2 | 107722668-107728939 | *IHH* | ENSBTAG00000008452 | Indian hedgehog |
| 2 | 123392015-123401324 | *MATN1* | ENSBTAG00000003479 | Matrilin 1, cartilage matrix protein |
| 2 | 131791680-131857642 | *ALPL* | ENSBTAG00000008951 | Alkaline phosphatase liver/bone/kidney |
| 2 | 7318227-7356937 | *COL3A1* | ENSBTAG00000021466 | Collagen, type III, alpha 1 |
| 2 | 94940392-94941459 | *GPR1* | ENSBTAG00000017043 | G protein-coupled receptor 1 |
| 3 | 40448699-40682012 | *COL11A1* | ENSBTAG00000021217 | collagen type XI alpha 1 chain |
| 3 | 21567426-21584444 | *ITGA10* | ENSBTAG00000008373 | integrin subunit alpha 10 |
| 3 | 95598417-95601695 | *RNF11* | ENSBTAG00000012996 | ring finger protein 11 |
| 3 | 43400326-43445130 | *SLC35A3* | ENSBTAG00000012454 | solute carrier family 35 member A3 |
| 3 | 15004847-15093527 | *GON4L* | ENSBTAG00000020356 | gon-4-like |
| 3 | 104065149-104084575 | *P3H1* | ENSBTAG00000017382 | prolyl 3-hydroxylase 1 |
| 3 | 106396860-106410914 | *COL9A2* | ENSBTAG00000020622 | Collagen, type IX, alpha 2 |
| 3 | 14603348-14604401 | *BGLAP* | ENSBTAG00000009433 | Bone gamma-carboxyglutamic acid (Gla) |
| 3 | 19994998-20007861 | *CTSK* | ENSBTAG00000021035 | Cathepsin K |
| 3 | 6672018-6847222 | *DDR2* | ENSBTAG00000004885 | Discoidin domain receptor tyrosine kinase 2 |
| 3 | 78601770-78644543 | *SLC35D1* | ENSBTAG00000005445 | Solute carrier family 35 (UDP-GlcA/UDP-GalNAc transporter), member D1 |

**Additional file 3 continued.**

| Bovine  chromosome | Position in bp  (UMD3.1 assembly) | Bovine gene | Gene ID | Gene name |
| --- | --- | --- | --- | --- |
| 3 | 9517705-9523145 | *PEX19* | ENSBTAG00000007537 | Peroxisomal biogenesis factor 26 |
| 4 | 9637833-9723444 | *PEX1* | ENSBTAG00000024431 | peroxisomal biogenesis factor 1 |
| 4 | 114435563-114450607 | *SLC4A2* | ENSBTAG00000011226 | solute carrier family 4 member 2 |
| 4 | 11624470-11661163 | *COL1A2* | ENSBTAG00000013472 | Collagen, type I, alpha 2 |
| 4 | 88250496-88354594 | *SLC13A1* | ENSBTAG00000006674 | Solute carrier family 13 |
| 5 | 32450453-32481246 | *COL2A1* | ENSBTAG00000013155 | collagen type II alpha 1 chain |
| 5 | 109855122-109867267 | *PEX26* | ENSBTAG00000011826 | peroxisomal biogenesis factor 26 |
| 5 | 103530536-103547919 | *PEX5* | ENSBTAG00000010490 | peroxisomal biogenesis factor 5 |
| 5 | 82241708-82258858 | *PTHLH* | ENSBTAG00000006538 | parathyroid hormone like hormone |
| 5 | 57639565-57643834 | *SUOX* | ENSBTAG00000006160 | sulfite oxidase |
| 5 | 106208179-106216757 | *FGF23* | ENSBTAG00000030343 | Fibroblast growth factor 23 |
| 5 | 26180271-26182515 | *HOXC8* | ENSBTAG00000012149 | Homeobox C8 |
| 5 | 86571273-87036285 | *SOX5* | ENSBTAG00000022360 | SRY (sex determining region Y)-box 5 |
| 5 | 95456444-95459983 | *MGP* | ENSBTAG00000012370 | Matrix Gla protein |
| 6 | 30797077-31322308 | *BMPR1B* | ENSBTAG00000002081 | bone morphogenetic protein receptor type 1B |
| 6 | 105170284-105275224 | *EVC* | ENSBTAG00000004287 | EvC ciliary complex subunit 1 |
| 6 | 105291556-105437262 | *EVC2* | ENSBTAG00000004277 | EvC ciliary complex subunit 2 |
| 6 | 109690990-109704041 | *FGFR3* | ENSBTAG00000007164 | fibroblast growth factor receptor 3 |
| 6 | 97652569-97735627 | *PRKG2* | ENSBTAG00000002978 | protein kinase, cGMP-dependent, type II |
| 7 | 63280729-63302599 | *SLC26A2* | ENSBTAG00000014615 | solute carrier family 26 member 2 |
| 7 | 13596367-13658112 | *NFIX* | ENSBTAG00000018229 | Nuclear factor I/X |
| 7 | 4354380-4361950 | *COMP* | ENSBTAG00000004630 | Cartilage oligomeric matrix protein |
| 7 | 85845448-85924686 | *HAPLN1* | ENSBTAG00000012411 | Hyaluronan and proteoglycan link protein 1 |
| 8 | 105095607-105203080 | *COL27A1* | ENSBTAG00000019807 | collagen type XXVII alpha 1 chain |
| 8 | 60381930-60386110 | ENSBTAG00000047598 | ENSBTAG00000047598 | − |
| 8 | 1266978-1394750 | *NEK1* | ENSBTAG00000026915 | NIMA-related kinase 1 |
| 8 | 60244066-60244343 | *RNase_MRP* | ENSBTAG00000042458 | RNA component of mitochondrial RNA processing endoribonuclease |

**Additional file 3 continued.**

| Bovine  chromosome | Position in bp  (UMD3.1 assembly) | Bovine gene | Gene ID | Gene name |
| --- | --- | --- | --- | --- |
| 9 | 34961718-34994278 | *COL10A1* | ENSBTAG00000003078 | collagen type X alpha 1 chain |
| 9 | 9516623-9608712 | *COL9A1* | ENSBTAG00000035054 | collagen type IX alpha 1 chain |
| 9 | 75813334-75891935 | *PEX7* | ENSBTAG00000016791 | peroxisomal biogenesis factor 7 |
| 9 | 30127786-30140793 | *GJA1* | ENSBTAG00000001835 | Gap junction protein, alpha 1 |
| 9 | 81967633-81999683 | *PEX3* | ENSBTAG00000001747 | Peroxisomal biogenesis factor 3 |
| 10 | 61876889-62142762 | *FBN1* | ENSBTAG00000002278 | fibrillin 1 |
| 11 | 78893508-78904614 | *MATN3* | ENSBTAG00000020893 | matrilin 3 |
| 11 | 77953380-78040118 | *APOB* | ENSBTAG00000008505 | apolipoprotein B |
| 11 | 106856171-106994803 | *COL5A1* | ENSBTAG00000047998 | Collagen, type V, alpha 1 |
| 11 | 43608812-43640302 | *PEX13* | ENSBTAG00000005257 | Peroxisomal biogenesis factor 13 |
| 11 | 47302536-47384700 | *EIF2AK3* | ENSBTAG00000000184 | Eukaryotic translation initiation factor 2 alpha kinase 3 |
| 12 | 12741069-12782474 | *TNFSF11* | ENSBTAG00000008924 | Tumor necrosis factor (ligand) superfamily, member 11 |
| 13 | 65340132-65343889 | *GDF5* | ENSBTAG00000004429 | growth differentiation factor 5 |
| 13 | 28254814-28275573 | *PHYH* | ENSBTAG00000007700 | Phytanoyl-CoA dioxygenase, peroxisomal |
| 13 | 20248945-20292114 | *ITGB1* | ENSBTAG00000015910 | Integrin, beta 1 |
| 13 | 75466513-75473824 | *MMP9* | ENSBTAG00000020676 | Matrix metallopeptidase 9 |
| 14 | 25544905-25560879 | *IMPAD1* | ENSBTAG00000015637 | inositol monophosphatase domain containing 1 |
| 14 | 1719732-1724221 | *SLC39A4* | ENSBTAG00000046026 | solute carrier family 39 member 4 |
| 14 | 42315041-42330314 | *PEX2* | ENSBTAG00000003885 | Peroxisomal biogenesis factor 2 |
| 15 | 77663790-77701218 | *LRP4* | ENSBTAG00000008429 | LDL receptor related protein 4 |
| 15 | 76816891-76823238 | *PEX16* | ENSBTAG00000003126 | peroxisomal biogenesis factor 16 |
| 15 | 36600420-37082361 | *SOX6* | ENSBTAG00000044185 | SRY (sex determining region Y)-box 6 |
| 15 | 39529628-39605792 | *FAR1* | ENSBTAG00000009061 | Fatty acyl CoA reductase 1 |
| 15 | 39728332-39730868 | *PTH* | ENSBTAG00000019080 | Parathyroid hormone |
| 15 | 52609012-52623088 | *INPPL1* | ENSBTAG00000019167 | Inositol polyphosphate phosphatase-like 1 |
| 15 | 5912037-5922736 | *MMP13* | ENSBTAG00000015059 | Matrix metallopeptidase 13 |
| 16 | 20592132-21286869 | *ESRRG* | ENSBTAG00000010392 | estrogen related receptor gamm |

**Additional file 3 continued.**

| Bovine  chromosome | Position in bp  (UMD3.1 assembly) | Bovine gene | Gene ID | Gene name |
| --- | --- | --- | --- | --- |
| 16 | 51683116-51688667 | *PEX10* | ENSBTAG00000009309 | Peroxisomal biogenesis factor 10 |
| 16 | 29150789-29177234 | *LBR* | ENSBTAG00000008453 | Lamin B receptor |
| 16 | 43848339-43992183 | *PEX14* | ENSBTAG00000013538 | Peroxisomal biogenesis factor 14 |
| 17 | 55898511-56018584 | *KDM2B* | ENSBTAG00000002328 | lysine demethylase 2B |
| 17 | 18553787-18573502 | *RAB33B* | ENSBTAG00000003208 | RAB33B, member RAS oncogene family |
| 17 | 65678454-65699847 | *TRPV4* | ENSBTAG00000000031 | Transient receptor potential cation channel, subfamily V, member 4 |
| 19 | 47689029-47748130 | *MRC2* | ENSBTAG00000015739 | mannose receptor C type 2 |
| 19 | 15081828-15086493 | *PEX12* | ENSBTAG00000019723 | peroxisomal biogenesis factor 12 |
| 19 | 48768617-48772049 | *GH1* | ENSBTAG00000017220 | growth hormone 1 |
| 19 | 11046574-11070478 | *TUBD1* | ENSBTAG00000001290 | tubulin delta 1 |
| 19 | 37088246-37104998 | *COL1A1* | ENSBTAG00000013103 | Collagen, type I, alpha 1 |
| 19 | 54020560-54024284 | *CANT1* | ENSBTAG00000009991 | Calcium activated nucleotidase |
| 19 | 59492677-59494539 | ENSBTAG00000045824 | ENSBTAG00000045824 | SRY (sex determining region Y)-box 9 |
| 20 | 58376939-58546811 | *ANKH* | ENSBTAG00000013391 | Mouse progressive ankylosis gene |
| 21 | 20800158-20869563 | ***ACAN*** | ENSBTAG00000016158 | aggrecan |
| 21 | 21133959-21198618 | *FANCI* | ENSBTAG00000009097 | Fanconi anemia complementation group |
| 21 | 7967701-8268340 | *IGF1R* | ENSBTAG00000021527 | insulin like growth factor 1 receptor |
| 21 | 70997217-71004742 | *PLD4* | ENSBTAG00000015160 | phospholipase D family member 4 |
| 21 | 57300674-57331304 | ENSBTAG00000026953 | ENSBTAG00000026953 | - |
| 22 | 53161300-53224114 | *PTH1R* | ENSBTAG00000016948 | parathyroid hormone 1 receptor |
| 22 | 21994945-22088653 | *SUMF1* | ENSBTAG00000039855 | Sulfatase modifying factor 1 |
| 22 | 43674720-43815706 | *FLNB* | ENSBTAG00000022004 | Filamin B |
| 22 | 7532626-7565025 | *CRTAP* | ENSBTAG00000021442 | Cartilage-associated protein |
| 23 | 13832465-13884536 | *MOCS1* | ENSBTAG00000010449 | molybdenum cofactor synthesis 1 |
| 23 | 16583122-16597438 | *PEX6* | ENSBTAG00000005532 | peroxisomal biogenesis factor 6 |
| 23 | 7290455-7318840 | *COL11A2* | ENSBTAG00000000601 | Collagen, type XI, alpha 2 |
| 24 | 49193606-49590504 | *DYM* | ENSBTAG00000000024 | Dymeclin |

**Additional file 3 continued.**

| Bovine  chromosome | Position in bp  (UMD3.1 assembly) | Bovine gene | Gene ID | Gene name |
| --- | --- | --- | --- | --- |
| 25 | 1627978-1666088 | ***PKD1*** | ENSBTAG00000020619 | Polycystic kidney disease 1 |
| 25 | 28630825-28636300 | *SBDS* | ENSBTAG00000004051 | Shwachman-Bodian-Diamond syndrome |
| 26 | 41823653-41926635 | *FGFR2* | ENSBTAG00000014064 | Fibroblast growth factor receptor 2 |
| 27 | 33250534-33291989 | *FGFR1* | ENSBTAG00000015457 | Fibroblast growth factor receptor 1 |
| 28 | 3378615-3418362 | *GNPAT* | ENSBTAG00000010773 | glyceronephosphate O-acyltransferase |
| 28 | 28254032-28256945 | *CHST3* | ENSBTAG00000011014 | Carbohydrate sulfotransferase-3 |
| 28 | 41817915-41875990 | *BMPR1A* | ENSBTAG00000000231 | Bone morphogenetic protein receptor, type IA |
| 29 | 47656594-47657616 | *FGF4* | ENSBTAG00000012563 | Fibroblast growth factor 4 |
| X | 139010588-139033622 | *ARSE* | ENSBTAG00000014932 | arylsulfatase E (chondrodysplasia punctata 1) |
| X | 138985621-139036733 | *ARSH* | ENSBTAG00000047161 | arylsulfatase family member H |
| X | 91735270-91739381 | *EBP* | ENSBTAG00000009287 | emopamil binding protein |
| X | 91994261-92013214 | *HDAC6* | ENSBTAG00000013244 | histone deacetylase 6 |
| X | 136987601-136999691 | *TRAPPC2* | ENSBTAG00000010922 | Trafficking protein particle complex 2 |
| X | 35387262-35413263 | *NSDHL* | ENSBTAG00000009231 | NAD(P) dependent steroid dehydrogenase-like |
| X | 39639906-39653687 | *BGN* | ENSBTAG00000005250 | Biglycan |
| X | 91735333-91739378 | *EBP* | ENSBTAG00000009287 | Delta(8)-delta(7) sterol isomerase emopamil-binding protein |
| X | 91994292-92013213 | *HDAC6* | ENSBTAG00000013244 | Histone deacetylase 6 |
