# Supplementary material for: A recessive lethal chondrodysplasia in a miniature zebu family results from an insertion affecting the chondroitin sulfat domain of aggrecan
Source: BMC Genet. 2018 Oct 11;19:91. doi: 10.1186/s12863-018-0678-8 (PMC6180608; doi:10.1186/s12863-018-0678-8)
Supplement: Supplementary file 8 — Primer pairs used for Sanger sequencing. Forward and reverse primers for validation of candidate variants found in ACAN and PKD1, amplicon size (AS), annealing temperature (AT) and number of cycles are shown. DNA derived from hair samples required an internal primer (ACAN_2). (DOCX 13 kb) [file 12863_2018_678_MOESM8_ESM.docx]

**Additional file 8** Primer pairs used for Sanger sequencing. Forward and reverse primers for validation of candidate variants found in *ACAN* and *PKD1*, amplicon size (AS), annealing temperature (AT) and number of cycles are shown. DNA derived from hair samples required an internal primer (*ACAN*_2).

| Primer pair | BTA | Gen | Polymorphism | Forward primers (5’-3’) | Reverse primer (5’-3’) | AT (°C) | Number  of cycles |
| --- | --- | --- | --- | --- | --- | --- | --- |
| *ACAN*_1 | 21 | *ACAN* | ACAN:g.20850999insC | CCCTCCGGAGCATATGACAG | TCGATTTCTAGACGCGCCTC | 60 | 26 |
| *ACAN*_2 | 21 | *ACAN* | ACAN:g.20850999insC | TTTTAGTGGGGACTTTTCTGGC | GGGACTGATGACACTTCTACCC | 58 | 42 |
| *PKD1*_1 | 25 | *PKD1* | PKD:g.1643626C>T | TCTTACACTTTCCGCTCTGTG | GTTCTTGGCCACTACTGTGAC | 58 | 42 |
