# Supplementary material for: A recessive lethal chondrodysplasia in a miniature zebu family results from an insertion affecting the chondroitin sulfat domain of aggrecan
Source: BMC Genet. 2018 Oct 11;19:91. doi: 10.1186/s12863-018-0678-8 (PMC6180608; doi:10.1186/s12863-018-0678-8)
Supplement: Supplementary file 9 — Primer pairs used for genotyping the ACAN:g.20850999insC variant. The single nucleotide candidate variant located in ACAN was genotyped using a Kompetitive Allele Specific PCR (KASP) assay. Forward and reverse primers, fluorescent labels (FAM/VIC), annealing temperature (AT) and number of cycles are shown. (DOCX 13 kb) [file 12863_2018_678_MOESM9_ESM.docx]

**Additional file 9** Primer pairs used for genotyping the *ACAN*:g.20850999insC variant. The single nucleotide candidate variant located in *ACAN* was genotyped using a Kompetitive Allele Specific PCR (KASP) assay. Forward and reverse primers, fluorescent labels (FAM/VIC), annealing temperature (AT) and number of cycles are shown.

| BTA | Gen | Polymorphism | Forward primers (5’-3’) | Reverse primer (5’-3’) | AT (°C) | Number  of cycles |
| --- | --- | --- | --- | --- | --- | --- |
| 21 | *ACAN* | ACAN:g.20850999insC | GGGGTGTATGTTACGGGGGGT-FAM  GGGTGTATGTTACGGGGGGG-VIC | TTACTGAACCAACYGTTTCCCAGGAA | 60 | 26 |
